# Effects of Hydrothermal Time on Structure and Photocatalytic Property of Titanium Dioxide for Degradation of Rhodamine B and Tetracycline Hydrochloride

**DOI:** 10.3390/ma14195674

**Published:** 2021-09-29

**Authors:** Mao Tang, Yangwen Xia, Daixiong Yang, Jiawei Liu, Xiaodong Zhu, Renyong Tang

**Affiliations:** 1 School of Mechanical Engineering, Chengdu University, Chengdu 610106, China; tangmao@cdu.edu.cn (M.T.); x1278704108@163.com (Y.X.); yangdaixiong1998@163.com (D.Y.); jiawei081999@163.com (J.L.); 2 School of Food and Biological Engineering, Chengdu University, Chengdu 610106, China

**Keywords:** TiO_2_, hydrothermal time, surface area, photocatalytic activity

## Abstract

Using butyl titanate and absolute ethanol as raw materials, TiO_2_ was prepared by a hydrothermal method with different hydrothermal times, and the influences of hydrothermal time on the structure and photocatalytic performance of TiO_2_ were investigated. The obtained samples were characterized by XRD, SEM, TEM, BET, PL and DRS, separately. The results show that TiO_2_ forms anatase when the hydrothermal time is 12 h, forms a mixed crystal composed of anatase and rutile when the hydrothermal time is 24 h, and forms rutile when the hydrothermal time is 36 h. With the extension of hydrothermal time, anatase gradually transforms into rutile and the surface area decreases. Although TiO_2_-24 h and TiO_2_-36 h show lower photoinduced charge recombination and higher light source utilization, TiO_2_-12 h exhibits the highest photocatalytic activity owing to its largest surface area (145.3 m^2^/g). The degradation degree of rhodamine B and tetracycline hydrochloride reach 99.6% and 90.0% after 45 min.

## 1. Introduction

Employing photocatalytic technology to degrade pollutants is an effective route for environmental governance. TiO_2_ has received extensive attention due to its advantages such as chemical stability, low cost, mild reaction conditions and high photocatalytic activity [1,2,3,4,5]. The crystal structure, crystallinity, surface morphology, specific surface area and optical property of TiO_2_ are closely related to the preparation method. The most commonly used methods are mainly sol–gel [6,7,8] and hydrothermal methods [9,10,11,12]. Zhu et al. [6] used sol–gel methods to synthesize TiO_2_ under calcination at 540 °C. The particles are spherical with a certain extent agglomeration and the surface area is 40 m^2^/g. It is convenient to control the morphology of photocatalyst by a hydrothermal method, which does not require high-temperature calcination and is conducive to obtaining a large surface area and high photocatalytic activity [13,14,15,16,17,18]. Esparza et al. [16] prepared nanostructured TiO_2_ by a hydrothermal method. The crystal grain size was 13 nm, and the particles were composed of nanotubes and nanosheets with a large surface area (269 m^2^/g). Methylene blue (MB) was completely degraded under UV light after 120 min. Zhu et al. [13] prepared Cu-doped TiO_2_ under the conditions of 200 °C for 12 h by hydrothermal method. It was found that the photocatalyst was anatase/rutile mixed crystal structure and the surface area was 73.9 m^2^/g. The decolorization degree of rhodamine (RhB) was 99.4% after 60 min. Nesic et al. [18]. prepared lanthanum and vanadium co-doped titanium dioxide by a microwave-assisted hydrothermal method. The samples showed high crystallinity and were all anatase structures. The specific surface area of 0.02V-2La/TiO_2_ was 125 m^2^/g, and the decolorization degree of RhB was more than 90% after 90 min.

Hydrothermal conditions will affect the crystal structure, surface morphology and specific surface area, thereby affecting the photocatalytic performance [19,20]. Lee et al. [19] studied the effects of hydrothermal temperature from 120 to 210 °C on the morphology and photocatalytic performance of TiO_2_. The results show that the particles gradually change from granular to nanotube with the increasing temperature and TiO_2_ prepared at 180 °C displays the best photocatalytic performance. Changing the ratio of reactants can also affect the structure and photocatalytic performance of the products [21,22]. Li et al. [21] prepared TiO_2_ by using different molar ratios of tartaric acid to TiCl_3_. When the tartaric acid: TiCl_3_ is 0.1, the photocatalytic activity is the highest.

In the present study, at the fixed hydrothermal temperature 200 °C, TiO_2_ photocatalysts were prepared with a hydrothermal time of 12, 24 and 36 h, respectively. The obtained samples were characterized by XRD, SEM, TEM, BET, PL, DRS and the degradation of RhB and tetracycline hydrochloride (TC) to study the effects of hydrothermal time on the crystal structure, morphology, specific surface area, optical property and photocatalytic performance of TiO_2_.

## 2. Experimental Section

### 2.1. Material Preparation

10 mL butyl titanate and 20 mL absolute ethanol were mixed to prepare solution A. Solution B was made of 30 mL deionized water, 2 mL hydrochloric acid and 2 mL polyethylene glycol, which was added to solution A dropwise. After stirring for 1 h, the mixture was transfer into a 100 mL hydrothermal reactor and kept at 200 °C for 12 h, 24 h and 36 h. After washing and drying, TiO_2_ photocatalysts were obtained. No further calcination process was performed. The samples obtained with different hydrothermal times were labeled as TiO_2_-12 h, TiO_2_-24 h and TiO_2_-36 h. 

### 2.2. Characterization

The crystal structure was characterized by DX-2700 X-ray diffractometer (XRD). Morphology was observed using Hitachi SU8220 scanning electron microscope (SEM) and FEI-Tecnai G2 F20 transmission electron microscope (TEM). Surface area was measured by an ASAP2460 surface area analyzer (BET). The optical property was studied using UV-3600 ultraviolet-visible spectrophotometer (DRS) and F-4600 fluorescence spectrometer (PL).

### 2.3. Photocatalysis Experiment

To achieve adsorption and desorption equilibrium, 0.1 g TiO_2_ powder and 100 mL (10 mg/L) RhB solution or 100 mL (30 mg/L) TC solution were mixed and then stirred 30 min in the dark. Using a 250 W xenon lamp as the light source, the mixture was taken every 15 min to measure the absorbance and the degradation degree was calculated by the formula (A_0_ − A_t_)/A_0_ × 100%.

## 3. Results and Discussion

### 3.1. Crystal Structure 

**Figure 1:**
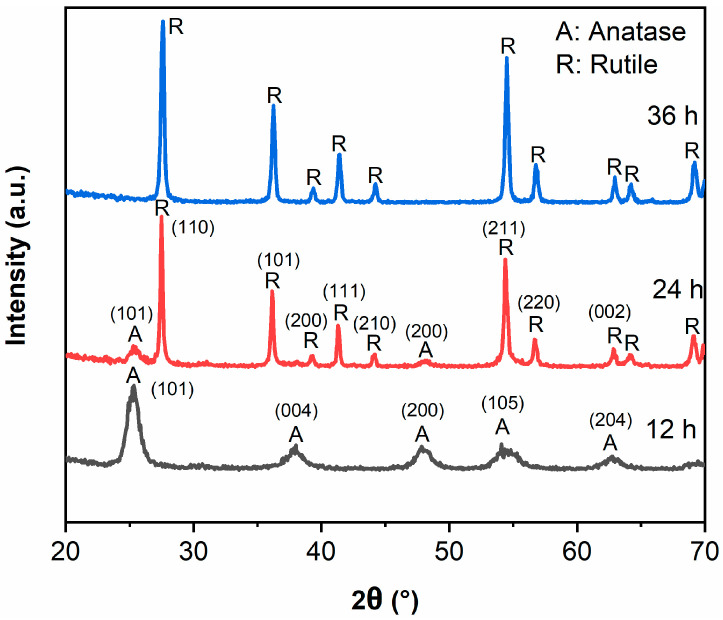
XRD patterns of TiO_2_-12 h, TiO_2_-24 h and TiO_2_-36 h.

Figure 1 exhibits the XRD patterns of samples. All peaks in TiO_2_-12 h correspond to anatase structure, indicating TiO_2_ forms anatase when the hydrothermal time is 12 h. The peak intensity of anatase (101) plane in TiO_2_-24 h drops sharply, and the peaks of anatase (004), (200), (105), (204) planes disappear. Only the peaks around 25.3 and 48.1° ascribing to the (101) and (200) crystal planes of anatase structure can be detected. Meanwhile, the rutile diffraction peaks appear and the intensity is much higher than anatase, implying that TiO_2_-24 h forms anatase/rutile mixed crystal structure. The mass fraction of anatase (X_A_) can be calculated by the following formula [2,21]:

X_A_ = (1 + 1.26(I_R_/I_A_))^−1^

where I_A_ and I_R_ represent the intensities of anatase (101) plane and rutile (110) plane, respectively. The mass fraction of anatase phase is 7.1% and the mass fraction of rutile phase is 92.9% in TiO_2_-24 sample. The anatase diffraction peaks in TiO_2_-36 h disappear wholly and all the peaks can be attributed to rutile, which indicates that the transformation from anatase to rutile has been completed when hydrothermal time is 36 h [23,24]. The grain sizes (D) of samples were calculated by the Scherrer formula [2]:

D = 0.89λ/βcosθ

where λ represents the wavelength of Cu Ka, β represents the full width at half maximum of the XRD peak ((101) plane for anatase and (110) plane for rutile), and 2θ represents the Bragg diffraction angle. The grain sizes (D) of TiO_2_-12, TiO_2_-24 and TiO_2_-36 are 9.9 nm, 13.2 nm (anatase)/35.0 nm (rutile) and 27.5 nm.

### 3.2. Morphology and Surface Area

**Figure 2:**
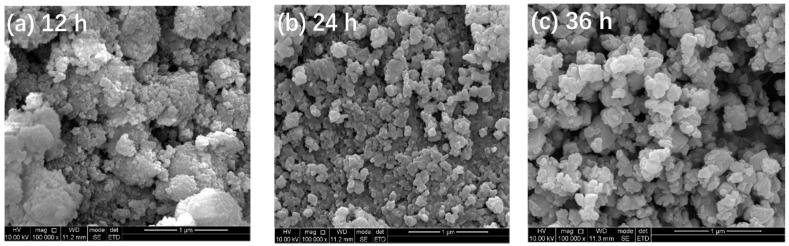
SEM images of TiO_2_-12 h (**a**), TiO_2_-24 h (**b**) and TiO_2_-36 h (**c**).

Figure 2 depicts the SEM images of TiO_2_-12 h, TiO_2_-24 h and TiO_2_-36 h. It is observed in Figure 2a that TiO_2_-12 h is composed of fine particles which further constitute agglomerates. The agglomerate size ranges from tens to hundreds of nanometers. The agglomeration of TiO_2_-24 h in Figure 2b is more obvious. In Figure 2c, the particles of TiO_2_-36 h are flaky and massive.

**Figure 3:**
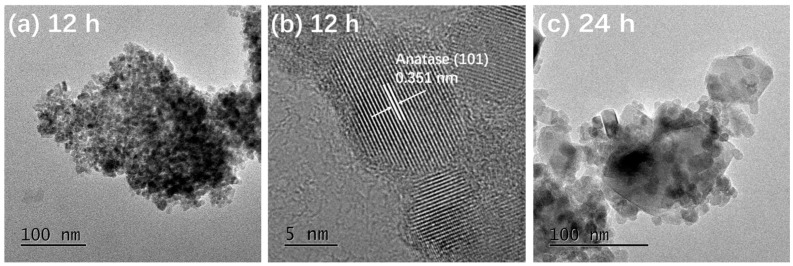

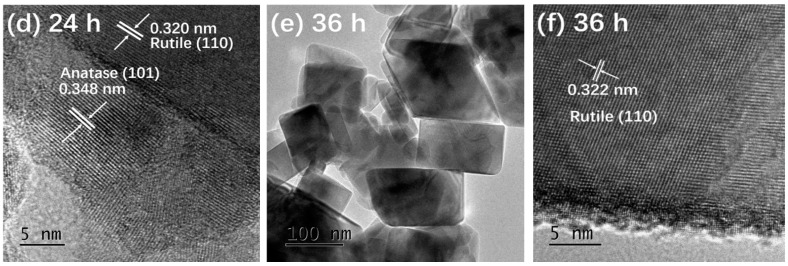
TEM and HRTEM images of TiO_2_-12 h (**a**,**b**) TiO_2_-24 h (**c**,**d**) and TiO_2_-36 h (**e**,**f**).

Figure 3 presents the TEM and HRTEM images of TiO_2_-12 h Figure 3a,b, TiO_2_-24 h Figure 3c,d and TiO_2_-36 h Figure 3e,f. In Figure 3a, the particles are relatively dispersed and the size of a single particle is around 10 nm. The interplanar spacing in Figure 3b is 0.351 nm, corresponding to the (101) crystal plane of anatase [5]. It is observed from Figure 3c that the size of a single particle is 15–30 nm, which is larger than that of TiO_2_-12 h. In addition to the granular shape, several particles exhibit rod and block shapes. The length of the nanorods is about 50 nm and the width is 15 nm. The size of the blocks is 50–100 nm. In Figure 3d, the marked interplanar spacing 0.348 nm corresponds to the (101) crystal plane of anatase and 0.320 nm corresponds to the (110) crystal plane of rutile [13,20], indicating that TiO_2_-24 h is a mixed crystal composed of anatase and rutile, which is in line with XRD results. Nanoparticle almost disappears in Figure 3e and the particles are completely made of rods and blocks. The length of rods is 100 nm and the width is approximately 20 nm. The size of the blocks is around 120 nm. The interplanar spacing marked in Figure 3f is 0.322 nm, corresponding to the (110) crystal plane of rutile.

**Figure 4:**
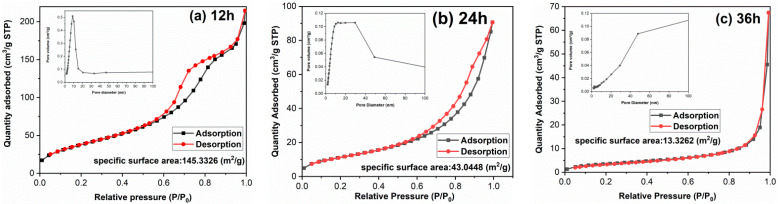
Nitrogen adsorption–desorption isotherms and pore size distribution curves of samples: (**a**) TiO_2_-12 h, (**b**) TiO_2_-24 h and (**c**) TiO_2_-36 h.

The morphology of TiO_2_ has a great impact on surface area and adsorption performance. It is found in Figure 3 that with the extension of hydrothermal time, the single particle size increases and the morphology changes significantly, which may lead to the surface area difference. To clarify the influences of hydrothermal time on surface area and the porosity of samples, the textural properties of samples have been implemented and the results are shown in Figure 4 and Table 1. Both TiO_2_-12 h and TiO_2_-24 h are mesoporous materials. The pore size distribution curve of TiO_2_-12 h shows a narrow peak, and its pore size distribution is uniform, and the pore size is between 5–15 nm. The pore size distribution curve of TiO_2_-24 h shows a broad peak shape, and the pore size distribution is uneven with a size of 5–50 nm. There is no peak in the pore size distribution curve of TiO_2_-36 h, indicating that no obvious mesopores can be detected in TiO_2_-36 h. The BET surface area, pore volume and average pore size of samples are summarized in Table 1. As the hydrothermal time increases, the BET surface area decreases from 145.3 m^2^/g to 43.0 m^2^/g and 13.3 m^2^/g, and the pore volume is reduced from 0.264 cm^3^/g to 0.107 cm^3^/g and 0.029 cm^3^/g. TEM images show that as hydrothermal time increases from 12 to 24 h, part of fine nanoparticles aggregate to form nanorods and nanoblocks. When the reaction time is 36 h, all the nanoparticles aggregate to form nanorods and nanoblocks. The agglomeration phenomenon is further intensified, and the BET surface area and pore volume are reduced.

Controlling the morphology of TiO_2_ and increasing its specific surface area is a research hotspot [25,26,27]. Du et al. [25] prepared porous Sn-doped TiO_2_ using polystyrene microspheres as a template. After calcination to remove the template, the porous structure was fabricated and the surface area reached 71.1 m^2^/g. Huang et al. [26] used a sol–gel method combined with a hydrothermal method to prepare TiO_2_ microspheres by a two-step reaction. The diameter of the microspheres was about 200–500 nm and the surface area was 91.1 m^2^/g. TiO_2_ nanospheres with diameter of 50–100 nm and surface area of 70.0 m^2^/g were fabricated by hydrothermal method in Mohamed et al.’s work [27]. In the present study, TiO_2_-12 h exhibits a relatively large surface area (145.3 m^2^/g), which may result in high photocatalytic activity.

### 3.3. Optical Property

**Figure 5:**
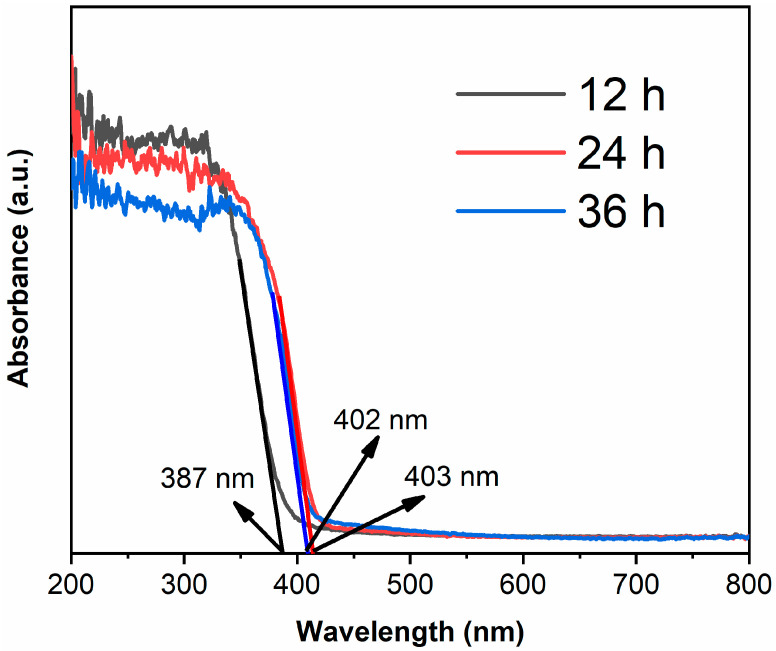
The UV-visible absorption spectra of TiO_2_-12 h, TiO_2_-24 h and TiO_2_-36 h.

Figure 5 shows the UV-visible absorption spectra of samples. The absorption edge of TiO_2_-12 h is 387 nm [28,29], which corresponds to anatase structure. The absorption edges are 403 and 402 nm for TiO_2_-24 h and TiO_2_-36 h, respectively. The band gap of rutile is smaller than anatase, thus TiO_2_-24 h and TiO_2_-36 h, which mainly consist of rutile, show red shift compared to TiO_2_-12 h.

**Figure 6:**
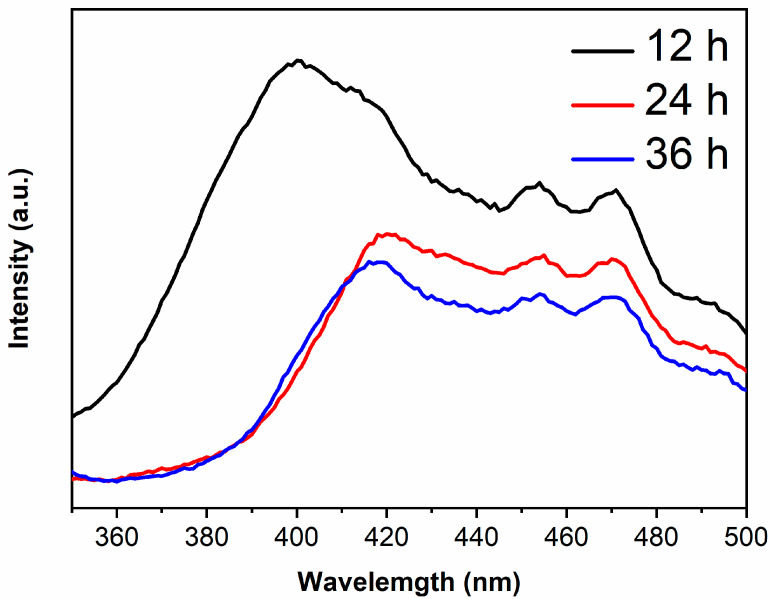
The PL spectra of TiO_2_-12 h, TiO_2_-24 h and TiO_2_-36 h.

The PL peaks are derived from the recombination of photogenerated electrons and holes, thus the lower peak intensity and the lower recombination rate [30,31]. The PL spectra of samples are shown in Figure 6. It is generally believed that the recombination rate of anatase is lower than rutile [32,33], however, the PL peak intensity of TiO_2_-12 h is the highest in the present work. XRD results show that the peak intensity of TiO_2_-12 h is low and the half-height width of peak is large, indicating that TiO_2_-12 h displays poor crystallinity with plentiful defects and oxygen vacancies. Photoinduced charges will be captured by defects and oxygen vacancies, which is in favor of retarding the recombination. However, excess defects or oxygen vacancies will introduce new recombination centers, enhancing the PL peak intensity [34]. With the increase in hydrothermal time, the crystallinity of the sample improves and the defects and oxygen vacancies reduce. Moderate defects and oxygen vacancies are beneficial to the separation of photogenerated electrons and holes [35,36]. Therefore, the PL peak intensity of TiO_2_-24 h and TiO_2_-36 h is lower than TiO_2_-12 h.

The PL main peak originates from photogenerated electrons in the conduction band returning directly to the valence band and recombining with holes, therefore, the wavelength corresponding to main peak in PL spectra is related to the band gap [37,38]. The main peak wavelengths of TiO_2_-12 h, TiO_2_-24 h and TiO_2_-36 h are 400, 415 and 415 nm, which are right shifted about 13 nm compared to their absorption edges due to the Stokes shift [39,40].

### 3.4. Photocatalytic Activity

**Figure 7:**
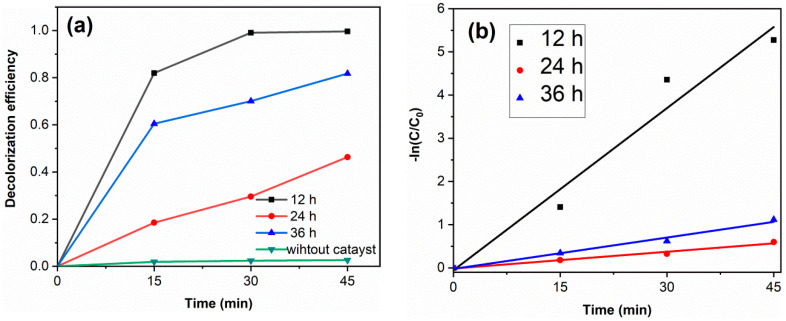
The RhB decolorization curves (**a**) and the kinetics fitting curves (**b**) of samples. (catalyst 0.1 g, RhB solution 100 mL (10 mg/L) and neutral pH).

Figure 7a shows the RhB decolorization curves of samples. Without catalyst, the decolorization degree of RhB is 2.6%, which indicates that the decolorization of RhB is mainly due to the degradation of photocatalysts. The decolorization degrees of TiO_2_-12 h, TiO_2_-24 h and TiO_2_-36 h are 99.6, 46.3 and 81.8% after 45 min. Figure 7b displays the kinetics fitting curves of samples. The apparent first-order rate constants k of TiO_2_-12 h, TiO_2_-24 h and TiO_2_-36 h are 0.125, 0.013 and 0.024 min^−1^, respectively. TiO_2_-12 h shows the highest photocatalytic activity. Although PL spectra and DRS spectra show that TiO_2_-24 h and TiO_2_-36 h exhibit a lower photogenerated charge recombination rate and higher visible light absorption, their photocatalytic activity is lower than that of TiO_2_-12 h yet. Morphology and BET results show that TiO_2_-12 h is composed of fine particles and possesses a relatively high surface area (145.3 m^2^/g), which is much higher than TiO_2_-24 h (43.0 m^2^/g) and TiO_2_-36 h (13.3 m^2^/g). A high surface area provides more reactive sites, thus TiO_2_-12 h shows the highest photocatalytic activity. Several RhB decolorization data reported by literatures via hydrothermal method are summarized in Table 2.

**Figure 8:**
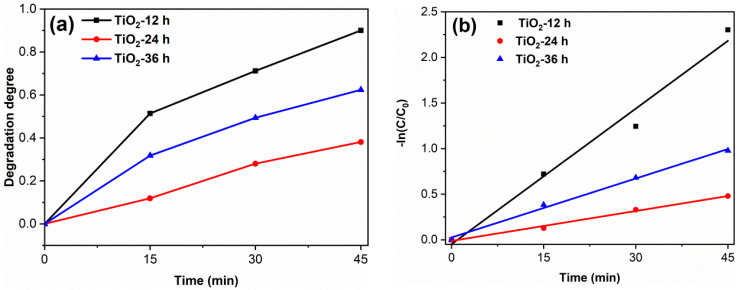
The TC degradation curves (**a**) and the kinetics fitting curves (**b**) of samples. (catalyst 0.1 g, TC solution 100 mL (30 mg/L) and neutral pH).

To study the degradation effect of the prepared photocatalyst on pharmaceutical waste, tetracycline hydrochloride (TC) was selected as the target pollutant. The results are shown in Figure 8. The degradation degree of TiO_2_-12 h, TiO_2_-24 h and TiO_2_-36 h are 90.0, 39.1 and 62.4%. The apparent first-order rate constants k of TiO_2_-12 h, TiO_2_-24 h and TiO_2_-36 h are 0.050, 0.011 and 0.022 min^−1^, respectively. The photodegradation results of RhB and TC confirm that TiO_2_-12 h is an efficient photocatalyst, which shows potential application prospects in the field of dye wastewater and pharmaceutical wastewater.

### 3.5. Photocatalytic Mechanism

**Figure 9:**
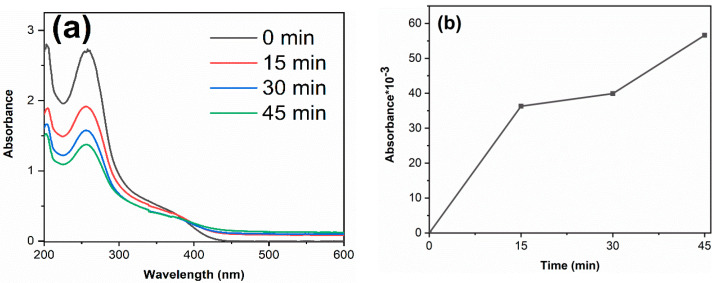
The absorbance curves of NBT (**a**) and 2, 3-HBA (**b**) of TiO_2_-12 h.

To verify the free radicals generated during the photocatalytic reaction, nitro-blue tetrazolium (NBT) and salicylic acid (SA) tests were carried out on TiO_2_-12 h sample. The detailed processes of NBT and SA experiments are as follows: Add 0.1 g TiO_2_-12 h powder into 100 mL NBT solution (0.05 mmol/L) and 100 mL SA solution (0.02 mol/L), respectively, keep stirring, and test their absorbance every 15 min after light irradiation. The results are shown in Figure 9. The photoinduced electrons are excited to conduction band and react with O_2_ to produce O_2_^−^ radicals, which further react with NBT. SA react with ·OH radicals, forming 2,3-HBA. Therefore, the decrease in NBT absorbance and the increased 2,3-HBA absorbance suggest that O_2_^−^ and ·OH radicals are generated under irradiation [48,49].

**Figure 10:**
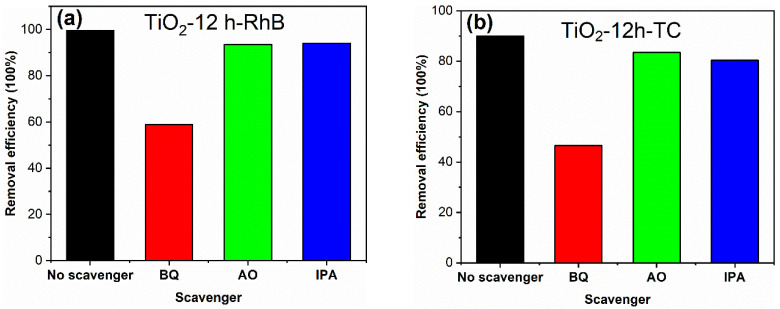
The degradation degrees of TiO_2_-12 h towards RhB (**a**) and TC (**b**) in the presence of different scavengers.

The active species in photocatalytic reaction process were investigated through adding benzoquinone (BQ), ammonium oxalate (AO) and isopropanol (IPA) as scavengers. The detailed processes of active species experiments are as follows: in the photocatalytic experiment, 2 mL (0.1 mol/L) BQ, AO and IPA solutions were added, respectively, keeping other test conditions unchanged. The results are shown in Figure 10. The decolorization degree of RhB for TiO_2_-12 h declines from 99.6 to 58.8, 93.6 and 94.0% in the presence of BQ, AO and IPA, respectively. Meanwhile, The degradation degree of TC for TiO_2_-12 h declines from 90.0 to 46.5, 83.4 and 80.3% in the presence of BQ, AO and IPA. Since BQ, AO and IPA capture ·O_2_^−^, h^+^ and OH species, it can be concluded that O_2_^−^ radicals are the main active groups in the degradation process, and h^+^ and OH radicals play a secondary role.

## 4. Conclusions

In summary, the influences of hydrothermal time on the structure and photocatalytic performance of TiO_2_ were studied systematically. TiO_2_-12 h forms anatase, TiO_2_-24 h forms anatase/rutile mixed crystal, and TiO_2_-36 h forms rutile. As the hydrothermal time increases, TiO_2_ gradually transforms from fine particles to lumps and the surface area decreases. TiO_2_-24 h and TiO_2_-36 h show a lower photogenerated charge recombination rate and higher visible light absorption, however, their photocatalytic activities are lower than that of TiO_2_-12 h, which can be attributed to the relatively high surface area (145.3 m^2^/g) of TiO_2_-12 h. Active species tests confirm that·O_2_^−^ radicals are the main active groups in the degradation process.

## Figures and Tables

**Table 1:** Textural properties of samples.

Samples	BET Surface Area (m^2^/g)	Pore Volume (cm^3^/g)	Average Pore Size (nm)
TiO_2_-12	145.3	0.264	7.26
TiO_2_-24	43.0	0.107	9.93
TiO_2_-36	13.3	0.029	8.74

**Table 2:** The RhB decolorization data of TiO_2_ photocatalytic materials prepared by hydrothermal method.

Refs	Method	Photocatalyst	Light Source	Decolorization Degree	k (min^−1^)
[10]	hydrothermal method	TiO_2_	Mercury lamp (500 W)	96.0% in 90 min	-
[13]	hydrothermal method	Cu-TiO_2_	Xenon lamp (250 W)	99.4% in 60 min	0.076 (RhB)
[17]	hydrothermal method	TiO_2_	Mercury lamp (450 W)	92.0% in 30 min	0.083 (RhB)
[41]	hydrothermal method	Ag-TiO_2_	Xenon lamp (800 W, >420 nm)	96.0% in 270 min	0.011 (RhB)
[42]	hydrothermal method	TiO_2_	Mercury lamp (300 W)	58.0% in 15 min	0.104 (RhB)
[43]	hydrothermal method	SDBS-TiO_2_	Xenon lamp (500 W)	90.0% in 120 min	0.0185 (RhB)
[44]	hydrothermal method	C-TiO_2_	Xe lamp (500 W, >400 nm)	94.3% in 120 min	0.022 (RhB)
[45]	hydrothermal method	Ag-TiO_2_	Xenon lamp (500 W)	80.0% in 240 min	-
[46]	hydrothermal method	Ag-TiO_2_	Xenon lamp (350 W)	100% in 45 min	-
[47]	hydrothermal method	ZnO-TiO_2_	Xenon lamp (350 W)	85.5% in 60 min	0.039 (RhB)
present work	hydrothermal method	TiO_2_	Xenon lamp (250 W)	99.0% in 30 min	0.125 (RhB)

## Data Availability

Data is contained within the article.
